# Low-Glycemic Index Diets as an Intervention in Metabolic Diseases: A Systematic Review and Meta-Analysis

**DOI:** 10.3390/nu14020307

**Published:** 2022-01-12

**Authors:** Chunxiao Ni, Qingqing Jia, Gangqiang Ding, Xifeng Wu, Min Yang

**Affiliations:** 1 The School of Public Health, Zhejiang University, Hangzhou 310027, China; nichunxiao@zju.edu.cn (C.N.); 13260930655@163.com (Q.J.); dinggq@chinacdc.cn (G.D.); xifengw@zju.edu.cn (X.W.); 2 National Institute for Nutrition and Health, Chinese Center for Disease Control and Prevention, Beijing 100050, China; 3 The Center of Clinical Big Data and Analytics, Second Affiliated Hospital, Zhejiang University School of Medicine, Hangzhou 310030, China

**Keywords:** glycemic index, metabolic disease, body mass, BMI, blood glucose, glycosylated hemoglobin, randomized controlled trial, meta-analysis, systematic review

## Abstract

We aimed to investigate the effects of a low-glycemic index (GI) diet on the body mass and blood glucose of patients with four common metabolic diseases by conducting a systematic review and meta-analysis of studies comparing a low-GI diet (LGID) and other types of diet. Search terms relating to population, intervention, comparator, outcomes, and study design were used to search three databases: PubMed, Embase, and the Cochrane Library. We identified 24 studies involving 2002 participants. Random-effects models were used for 16 studies in the meta-analysis and stratified analyses were performed according to the duration of the intervention. The systematic review showed that LGIDs slightly reduced body mass and body mass index (BMI) (*p <* 0.05). BMI improved more substantially after interventions of >24 weeks and there was no inter-study heterogeneity (I^2^ = 0%, *p* = 0.48; mean difference (MD) = −2.02, 95% confidence interval (CI): −3.05, −0.98). Overall, an LGID had superior effects to a control diet on fasting blood glucose (FBG) and glycosylated hemoglobin. When the intervention exceeded 30 days, an LGID reduced FBG more substantially (MD = −0.34, 95% CI: −0.55, −0.12). Thus, for patients with metabolic diseases, an LGID is more effective at controlling body mass and blood glucose than a high-GI or other diet.

## 1. Introduction

The prevention and treatment of metabolic diseases, such as diabetes mellitus, cardiovascular disease, obesity, and the metabolic syndrome, are a global public health issue. Between 1990 and 2019, the number of patients with cardiovascular disease increased from 271 million to 523 million and the number of associated deaths increased from 12.1 million to 18.6 million [1]. The age-standardized disability-adjusted life year (DALYR) for diabetes increased by 24.4% between 1990 and 2019 [2]. By the year 2030, the global cost of managing diabetes is expected to reach USD 2.1 trillion [3]. According to the International Diabetes Federation (IDF) Diabetes Atlas, the global prevalence of diabetes is expected to increase to 10.4% by 2040 [4]. Without adjusting for secular trends, the number of obese adults is expected to reach 573 million by 2030, and if the long-term trends continue unabated, the number of individuals with obesity is projected to total 1.12 billion [5]. The global prevalence of metabolic syndrome is ~25%, meaning that >2 billion people worldwide are now affected [4]. These common metabolic diseases involve disorders in multiple metabolic pathways, including insulin signaling, insulin secretion, glucose utilization, thermogenesis, mitochondrial function, and autophagy [6] and are all related to obesity [7].

The glycemic index (GI) was first developed by Jenkins et al. in the early 1980s [8] and is an index of the effect of the consumption of carbohydrate-containing foods on postprandial glucose concentrations. GI is defined as the ratio of the area under the glucose curve (AUC) 2 h after consuming 50 g of carbohydrates in test food vs. that for standard food. In general, glucose or white bread is used as the reference food. The formula used is:

$$\mathrm{GI}\;=\left(\mathrm{AUC}\;\mathrm{test}\frac{\mathrm{food}}{\mathrm{AUC}}\mathrm{reference}\;\mathrm{food}\right)\;\times \;100.$$

According to their GI, foods are divided into three categories: high-GI foods (GI≥70), medium-GI foods (55 ≤ GI < 70), and low-GI foods (GI < 55) [9,10,11].

The total carbohydrate intake in a mixed meal also directly affects the glycemic response, which has given rise to the concept of glycemic load (GL). GL can be calculated using the following formula:

$$\mathrm{GL}\;=\;\mathrm{GI}\;\times \mathrm{available}\;\mathrm{carbohydrate}\;\mathrm{in}\;\mathrm{the}\;\mathrm{food}\;\mathrm{item}\;\left(g\right)/100.$$

GL is also categorized as low (<10), intermediate (11–19), or high (>20) [9].

When a mixed meal contains carbohydrates from several different sources, the effect of the low-GI component is diluted proportionately by the others. Therefore, the GI of a mixed diet can be calculated [12]. At present, there are two methods used to obtain the GI of a diet: one is to measure dietary GI directly and the other is to calculate it using the GI of the components. For the latter, the proportion of carbohydrate in each component is multiplied by the GI of each to obtain the contributions of each component, which are added together to obtain the diet’s overall GI. Previous studies have shown that dietary GI values obtained using these two methods have high validity and that both can be used to accurately estimate the blood glucose response to a mixed diet [13].

Previous studies of the effects of low-GI diets (LGIDs) on metabolic diseases have generated inconsistent results. The study by Abete et al. showed that the effect of an LGID on weight loss was superior to that of a control diet [14], whereas the study by Mulyar et al. showed no difference [15]. The findings of studies by Hu et al. and Heilbronn et al. regarding the effects of LGIDs on blood glucose control also yielded opposing findings [16,17]. Single studies are often limited by their small sample size, differences in the characteristics of the participants, and sampling errors. In addition, the systematic reviews of the use of LGIDs published to date have focused on a single disease or only included observational studies. For example, those by Ojo et al. and Thomas et al. focused on diabetes alone [18,19,20,21,22,23] and those by Askari et al., Livesey et al., and Mirrahimi et al. only included observational studies [18,22,23].

In the present study, we aimed to compare the effects of an LGID and other diets (American Diabetes Association diet, Mediterranean diet, low-glycemic index diet, modified Mediterranean diet, high-glycemic index diet, low-fat diet, low-glycemic index Mediterranean die, low-carbohydrate diet, high-cereal fiber diet, routine diet, low-glycemic load diet, high-glycemic load diet) on weight loss and glycemic control in patients with one of four common metabolic diseases (obesity, metabolic syndrome, diabetes mellitus, and cardiovascular disease) by performing a systematic review and meta-analysis of randomized controlled trials (RCTs).

## 2. Materials and Methods

### 2.1. PRISMA Guidelines

We report the present findings in accordance with the PRISMA statement for reporting systematic reviews and meta-analyses of studies that evaluate healthcare interventions [24].

### 2.2. Data Sources and Search Strategy

A comprehensive search strategy was designed for PubMed and modified for use in other databases. The search strategy was:

((‘glycemic index’ OR ‘glycemic indices’ OR ‘glycemic index number*’ OR ‘glycaemic index ’OR ‘glycaemic indices’ OR ‘glycaemic index number*’) OR (‘glycemic load’ OR ‘glycaemic load’) OR ‘low-GI’ OR ‘high-GI’)

AND

(child OR children OR adolescent OR adolescence OR teenagers OR teenager OR youth OR adult OR elderly)

AND

(‘metabolic disease’ OR diabetes OR pre-diabetes OR ‘diabetes mellitus’ OR ‘cardiovascular disease’ OR ‘cardiovascular diseases’ OR obesity OR ‘metabolic syndrome’ OR ‘metabolism syndrome’ OR ‘metabolic syndromes’)

AND

(‘randomized controlled trial’ OR RCT)

AND

(diet OR dietary)

We searched the PubMed, Cochrane Library, and Embase databases. We did not restrict our search results by time or language. The final search was performed on 30 January 2021.

### 2.3. Eligibility Criteria

Studies were included if they met the following inclusion criteria: (1) the studies were RCTs conducted in children, adolescents or adults; (2) the participants had at least one of the following diseases: obesity, diabetes, metabolic syndrome, or cardiovascular disease; (3) the intervention was a diet that lowered or aimed to lower dietary GI and the control group consumed a high-GI, low-fat, low-carbohydrate, conventional weight-loss, or specialty diet; and (4) the study included data regarding body composition (e.g., body mass, BMI, or body fat content) or indices of blood glucose control (e.g., fasting blood glucose (FBG) or fasting insulin concentrations).

### 2.4. Study Selection

Abstracts and full-text articles were selected independently by two authors. Before further reading of each publication, neither researcher knew the results of the screening by the other. Disagreements were resolved by discussion, if necessary, with a third author.

### 2.5. Study Quality

Study quality was determined using the Cochrane Collaboration’s tool for assessing the risk of bias in randomized trials [25]. This tool evaluates the risk of bias in randomized trials in six domains: selection bias, performance bias, detection bias, attrition bias, reporting bias, and other bias. The study quality was assessed individually by two authors, and disagreements were settled by discussion, if necessary, with a third author.

### 2.6. Data Extraction

Data extraction was performed independently by two authors and the output was checked jointly. The data extracted included the basic characteristics of the study, the RCT-related characteristics, outcomes of interest, and study quality. We extracted the following data from each eligible article: the first author’s name, year of publication, country where the trial was performed, disease type, sample size, sex ratio, BMI, BMI category, mean age, FBG, glycosylated hemoglobin (HbA1c%), duration of the intervention, study length, randomization method, presence of blinding, energy restriction, intervention type, nature of the control group, GI, and percentage of carbohydrate consumed for the invention and control groups.

### 2.7. Statistical Synthesis and Analysis

Data from the included studies were subjected to meta-analysis using R version 4.1.0 (https://cloud.r-project.org/, accessed on 18 May 2021). This analysis was conducted to summarize the effects of an LGID on body mass, BMI, fasting glucose, and HbA1c% in individuals with diabetes, cardiovascular disease, obesity, or the metabolic syndrome. Heterogeneity testing was performed to evaluate the variability in the effects of the intervention [26]. If *p* < 0.05, or I^2^ > 40%, it was considered that there was significant heterogeneity in the data, and a random-effects model was selected. Otherwise, it was considered that the data were homogeneous. The mean differences (MD) in parameters were used in fixed-effect models, alongside 95% confidence intervals (CIs), and an α of 0.05.

## 3. Results

### 3.1. Results of the Literature Searches

**Figure 1:**
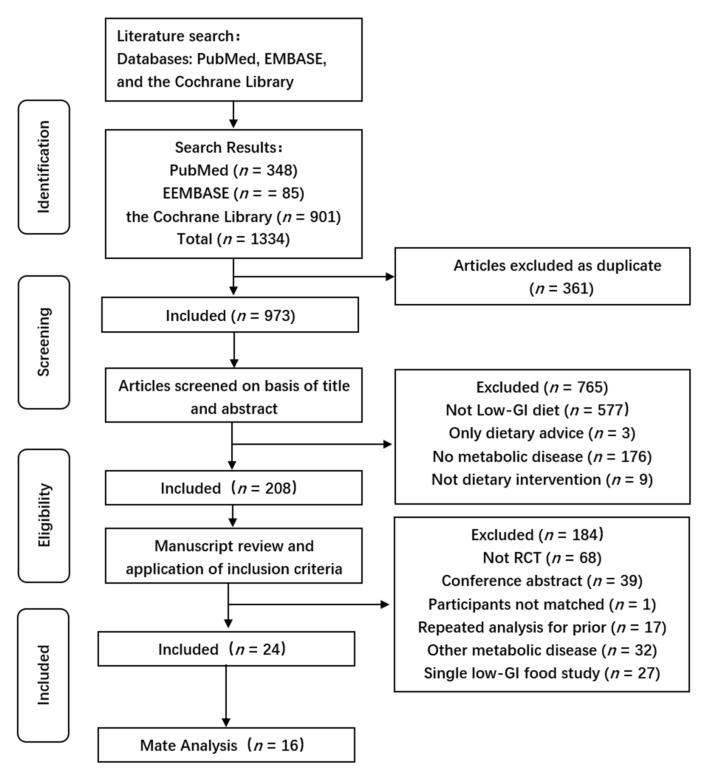
PRISMA flow diagram. Of an initial 1334 independent records, 361 were duplicates. A further 765 and 184 were excluded after scrutinization at the title/abstract and full-text levels, respectively, leaving 24 articles. Data from all 16 articles were included in the meta-analyses.

A flow chart describing the selection process and the detailed methods of study identification is presented in Figure 1. The primary search of the three databases yielded 1334 articles. Of these, 361 were duplicates, leaving 973 articles. The titles and abstracts of each were then scrutinized and 208 appropriate articles were selected. The full text inclusion criteria were applied to these, which yielded 24 articles for inclusion in the present analysis.

### 3.2. Characteristics of the Included Studies

The characteristics of the included studies are summarized in Table 1. The year of publication ranged from 2002 to 2020. Most of the studies were conducted in the United States and two in China. There were 12 studies of patients with type 2 diabetes, one of gestational diabetes, two of metabolic syndrome, and nine of obesity. The sample sizes of the studies ranged from 19 to 210, and except for those that included multiple control groups, the sample sizes of the intervention and control groups were relatively balanced. Two of the studies were of women alone, while the sex distribution of the others was more even. The BMIs of the participants ranged from 24.3 to 36.3 kg/m^2^. The average age ranged from 12 to 66.2 years. Only half of the included articles provided baseline fasting glucose concentrations, which ranged from 5.0 mmol/L to 10.3 mmol/L.

Table 2 shows the RCT-related characteristics and results of the quality evaluation of the included studies. The durations of the studies ranged from 3 months to 4 years and the durations of the interventions ranged from 5 days to 1 year. Most of the articles did not state the method of randomization used, but four were randomized using random numbers, two were randomized by subject ID, and two were randomized using opaque envelopes. Only five of the 24 studies used blinding and six used an energy restriction method. The diet of the intervention group was an LGID, except in one study, in which it was a low-GL diet. The mean GI of the diet in the intervention group was 36.1, which met the requirements for a low-GI diet [45]. The control diets consisted of the American Diabetes Association (ADA) Diet, a Mediterranean diet (Med-D), a modified Mediterranean Diet (MMD), a high-GI diet (HGID), a low-fat diet (LFD), a low-GI Mediterranean diet (LGIMD), a low-carbohydrate diet (LCHOD), a high-cereal fiber diet (HCFD), a routine diet (RD), or a high-GL diet (HGLD). Fraser et al. used MMD and the ADA Diet as dual controls [37], and Osela et al. used Med-D, LGIMD, and RD as triple controls [34]. In two of the studies, HGID and LCHOD were consumed by the control group [30,31]. The GIs of the diets consumed by the intervention groups ranged from 35.8 to 69.6, and the GIs of five of the studies were >55, which is consistent with a medium-GI diet. The GIs of the control diets were between 59 and 83.5, all of which were medium or high GIs. The percentage of total energy yielded by the carbohydrates (CHO%) ranged from 44% to 59.8% in the intervention group and 42.7% to 60.8% in the control group.

### 3.3. Quality of the Included Studies

**Figure 2:**
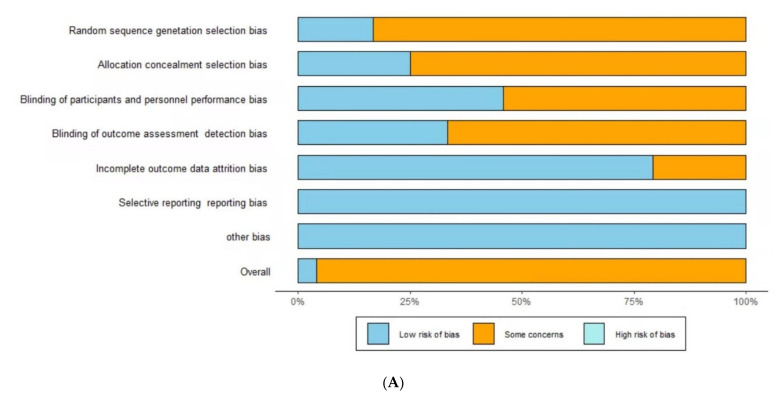

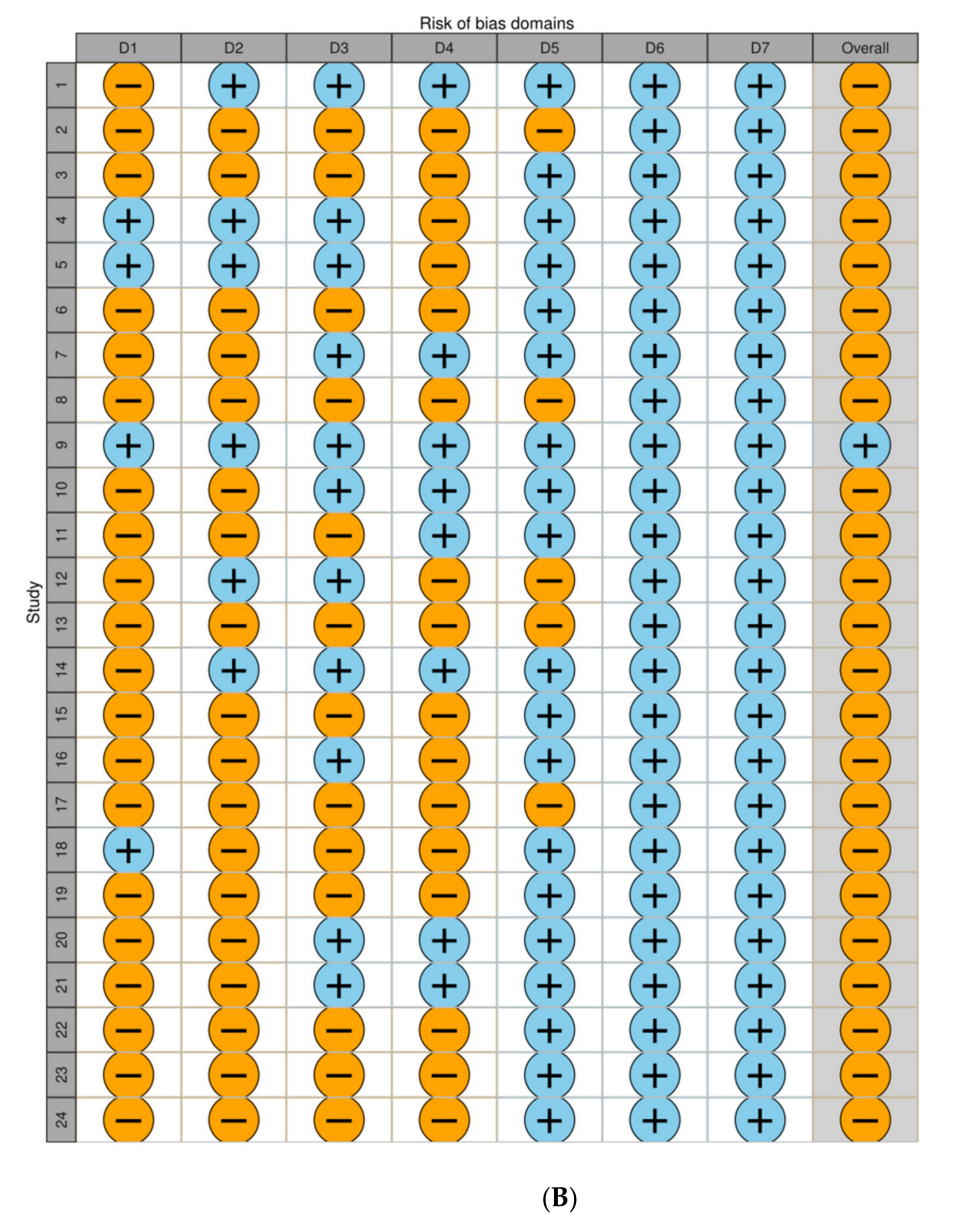
Risks of bias for the included studies. (**A**): Risk of bias graph; (**B**): risk of bias summary. D1 = Random sequence generation (selection bias), D2 = Allocation concealment (selection bias), D3 = Blinding of participants and personnel (performance bias), D4 = Blinding of outcome assessment (detection bias), D5 = Incomplete outcome data (attrition bias), D6 = Selective reporting (reporting bias), D7 = other bias. Sky blue (+) = low risk of bias, Orange (−) = unclear risk of bias, Turquoise (×) = high risk of bias.

The quality of the included studies was determined with reference to the Cochrane Collaboration Randomized Trials Bias Assessment tool [25]. The overall quality assessment of the included studies was “unclear risk” (Figure 2). Only one of the included studies was rated as low risk; the others were rated as unclear risk.

### 3.4. Body Mass

**Figure 3:**
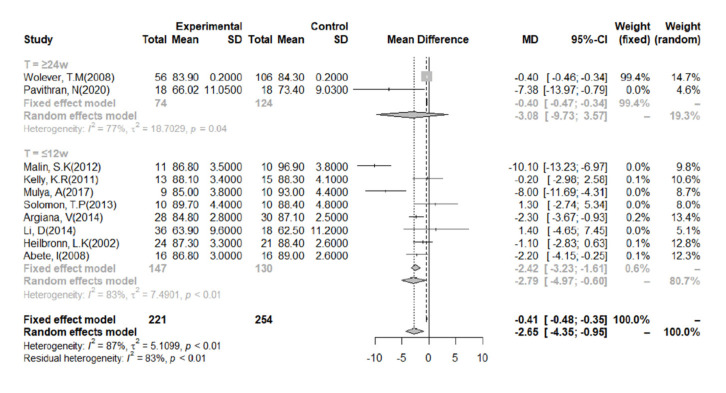
Meta-analysis of the effect of a low-glycemic index diet on body mass, according to the duration of the intervention. A random-effects model and mean difference analysis were used.

Eleven studies aimed to determine the effect of LGID interventions on body mass. We conducted a meta-analysis of the data after categorization according to the duration of the intervention (Figure 3). We found that a 7-day intervention had no significant effect on body mass; therefore, this was not included in the analysis [36]. Owing to the heterogeneity of the study data, a random-effects model was adopted. The combined data from all the included studies indicated that the LGIDs were superior to the other diets with respect to their body mass-lowering effect (I^2^ = 87%). The overall effect size (MD) was −2.65 (95% CI: −4.35, −0.95). Categorization of the data according to the duration of the intervention revealed a slight reduction in the heterogeneity of the studies when the duration was ≥24 weeks in length (I^2^ = 77%).

### 3.5. BMI

**Figure 4:**
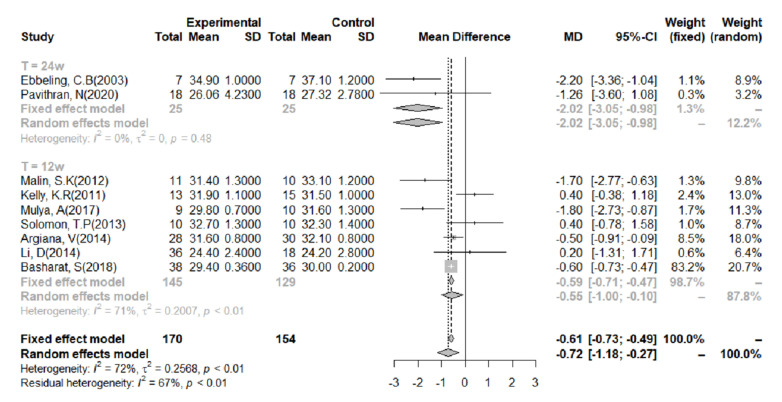
Meta-analysis of the effect of a low-glycemic index diet on body mass index, using data categorized according to the duration of the intervention. A random-effects model and mean difference analysis were used.

The relationship between BMI and an LGID was evaluated using data from 10 studies. One of the studies utilized a 7-day intervention, which we did not think would be sufficient to cause a significant change in BMI. Therefore, this study was not included in the meta-analysis [32]. The heterogeneity of the studies was very clear; therefore, a random-effects model was adopted (I^2^ = 72%; Figure 4). Overall, an LGID was found to be beneficial for BMI (MD = −0.72; 95% CI: −1.18, −0.27). A meta-analysis was then performed on the data after categorization according to the duration of the intervention. After a 24-week intervention, the heterogeneity of the studies was low and the intervention was shown to reduce BMI (I^2^ = 0%, MD = −2.02, 95% CI: −3.05, −0.98). When the intervention lasted for 12 weeks, the analysis also showed that an LGID was superior to the other diets at reducing BMI, although there was significant heterogeneity (I^2^ = 71%, MD = −0.55, 95% CI: −1.01, −0.10).

### 3.6. FBG

**Figure 5:**
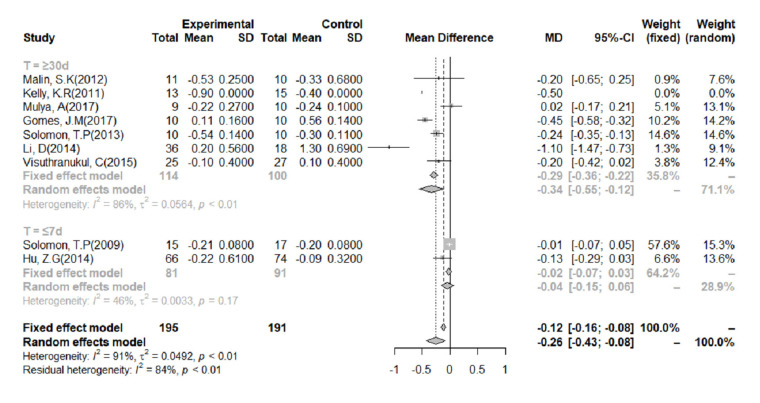
Meta-analysis of the effect of a low-glycemic index diet on fasting blood glucose concentration, categorized according to the duration of the study. A random-effects model and mean difference analysis were used.

FBG was measured in eight studies. We performed a meta-analysis of data from these studies, categorized according to the duration of the intervention (Figure 5). Because of the significant heterogeneity of the studies (I^2^ = 91%), we used a random-effects model. Overall, the analysis showed that an LGID was superior to a control diet at reducing FBG (MD = −0.26, 95% CI: −0.43, −0.08). The subgroup analysis showed that when the duration of the intervention was ≤7 days, there was no significant difference in the change in FBG between the LGID and control groups (MD = −0.04; 95% CI: −0.15, −0.06). However, when the intervention lasted ≥30 days, the LGIDs were found to be superior to the control diets at reducing FBG (MD = −0.34, 95% CI: −0.55, −0.12).

### 3.7. HbA1c%

**Figure 6:**
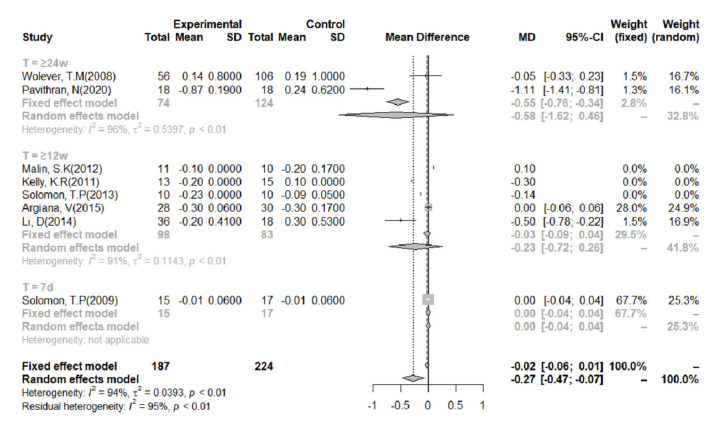
Meta-analysis of the effect of a low-glycemic index diet on glycated hemoglobin (HbA1c%), according to the duration of the intervention. A random-effects model and mean difference analysis were used.

Eight studies reported HbA1c% concentrations (Figure 6), and there was a high level of heterogeneity in these studies (I^2^ = 94%). Overall, the effect of LGIDs on HbA1c% was statistically significant (MD = −0.30, 95% CI = −0.49, −0.10). Meta-analysis of the data, categorized according to the duration of the intervention, showed that when it lasted for ≥24 weeks, the LGIDs tended to be superior to other diets at reducing HbA1c%, although the difference was not statistically significant (MD = −0.58, 95% CI = −1.62, 0.46).

## 4. Discussion

The present systematic review and meta-analysis show that an LGID is superior to a number of other diets in the control of body mass, which is consistent with the results of previous studies [15,32,40,43,47]. Furthermore, stratified analyses of the duration of the intervention showed that studies with a duration of ≥24 weeks generated more consistent results and larger reductions in body mass and BMI.

A previous systematic review and meta-analysis of RCTs performed in children, adolescents, or adults suggested that the effect of LGIDs on body mass takes at least 4 weeks to manifest [48]. Another meta-analysis of long-term (≥6 months) LGID/LGLD interventions showed that these reduce body mass by 0.62 kg more than a high-GI or other diet. In addition, it was shown that a long-term LGID/LGLD intervention is beneficial for the control of C-reactive protein and fasting insulin concentrations [21]. However, these two meta-analyses only included studies of overweight or obese people [21,48]. A study conducted in postpartum women with obesity showed that 12 weeks of consumption of an LGID reduces body mass and BMI, without reducing fat mass or muscle mass, compared to a control diet [46]. In a further RCT, a low-GL diet or control diet was assigned to 86 overweight or obesity adults. The trial was divided into two phases: the first was a 12-week initial weight-loss phase and the second was a 24−36-week weight maintenance phase. During the first stage, the weight loss-inducing effect of the low-GL diet was significantly better than that of the control diet, whereas during the second stage, there was no difference in weight loss between the two groups [49]. To gain further insight into the effects of such diets, more long-term RCT studies of LGID interventions are required.

We have also shown that an LGID is more effective than HGID or other diets at controlling FBG and HbA1c, as previously shown [19,50,51]. Subgroup analyses showed that LGID interventions of ≥4 weeks reduced FBG and HbA1c% more substantially than an HGID or other diet. Conversely, a 12-month RCT conducted in patients with type 2 diabetes did not show that LGID is better than a low-carbohydrate diet or an HGID at controlling HbA1c [31]. Previous studies have shown that postprandial hyperglycemia may account for 70% of the total daytime hyperglycemia and that the contribution of postprandial blood glucose fluctuations varies as blood glucose control deteriorates, which may explain the inconsistent relationship identified between a low-GI diet and HbA1c in previous studies [11]. The results of the present systematic review are consistent with those of a systematic review performed in 2018, which showed that an LGID is more effective at controlling HbA1c and FBG than an HGID or other diets. However, this previous review focused on adults with type 2 diabetes and did not involve an analysis that considered the duration of the intervention [19]. A study of LGID in children with diabetes showed that it is superior to a control diet at controlling HbA1c and also that the number of children consuming an LGID that achieve an acceptable HbA1c after 12 months is twice that of the number of children consuming a control diet [52]. Therefore, more RCT studies of >4 weeks’ duration should be conducted with FBG and HbA1c as outcomes, in order to collect more robust data in this field.

There was high heterogeneity in the studies that assessed all of HbA1c%, FBG, body mass, and BMI, which may suggest that previous studies of low-GI diets have not been well standardized. Food GI and the blood glucose response are also affected by the cooking method and time, properties of starch, particle size, pH, fiber content, fat content, and protein content, which can have significant effects on study outcomes [53]. One study of the effect of a low-GI diet on diabetes showed that it significantly reduced FBG vs. the habitual diet, a ‘healthy’ diet, an anti-hypertensive diet, and a low-carbohydrate diet [54]. However, because only 16 studies were included in the present analyses but eight different control diets were used, it is not possible to analyze the data according to the identity of the control diet. Furthermore, heterogeneity may be introduced because of differences in the classification of an LGID and an HGID in the literature [12,55]. In addition, most previous reports did not describe the proportions of carbohydrate, fat, and protein present in the diets used. This may explain why there has been inconsistency and controversy over the use of GI as a guide for the selection of foods by people with diabetes, and also why previous studies of the effects of an LGID on health and health-related outcomes have generated mixed results [12]. The Chinese Medical Nutrition Treatment Guidelines for Overweight/Obesity (2021) include an LGID as one of the diets that are suitable for the treatment of overweight and obesity and indicates that such a diet is beneficial with respect to weight loss, satiety, and insulin resistance, which is consistent with the results of the present study. However, the guidelines do not clearly define an LGID, nor do they specify appropriate ranges for the contents of the three macronutrients in an LGID [56]. Therefore, an explicit definition of an LGID is urgently required, so that more comprehensive and repeatable research regarding the use of LGIDs can be performed.

An important reason for claiming that LGID is beneficial to overall health is that LGID may help with weight control because it can promote satiety. Another important reason is that LGID can stimulate a sustained and small release of insulin by slowly releasing glucose into the bloodstream, causing a slow increase in blood glucose and insulin concentrations over time, flattening the postprandial blood glucose and insulin curves [48,57]. It is worth noting that in addition to body weight and BMI, there are more indicators, such as resting energy expenditure, body fat percentage and cholesterol, etc., which can indicate the effect and cause of weight loss, as well as other health effects. A randomized controlled trial study conducted in obese or overweight people found that LGID can promote resting and total energy expenditure [58]. A previous review showed that a LGID may reduce high-density-lipoprotein cholesterol and triacylglycerol levels [59]. Unfortunately, these metrics are relatively expensive and difficult to obtain. Therefore, there are currently relatively few articles using indicators such as resting energy and body fat percentage. In the future, further research can be carried out based on the above indicators.

### Strengths and Limitations of the Study

We have used a systematic review and meta-analysis to provide more comprehensive evidence of the positive effects of low-GI diets on body mass, BMI, FBG, and HbA1c% than obtained previously. All the included studies were RCTs without a unilateral bias and the quality of evidence obtained was assessed using the Cochrane Collaboration tool. Seventeen RCTs were selected using the PICOS principles. None of these trials were rated as having a significant risk of bias and there was no evidence of publication bias.

There were several limitations to the present study. First, relatively few studies were included in the meta-analysis and the sample size of most of these was relatively small. Second, there was considerable heterogeneity in these studies, which may be associated with the large differences between studies with respect to the baseline values of parameters and the durations of the interventions. Analysis of categorized data did not significantly reduce this heterogeneity. Third, the method of data extraction was non-blinded, which represents a potential source of bias. Fourth, some linguistic bias is unavoidable, and the only reports identified were written in English. Finally, most of the constituent studies did not report their findings according to sex; therefore, we could not determine whether there was an influence of sex on the outcomes.

## 5. Conclusions

The present systematic review and meta-analysis provide evidence that an LGID is superior to an HGID or other diet for the control of body mass, BMI, FBG, and HbA1c% in patients with one of four common metabolic diseases (obesity, metabolic syndrome, diabetes and cardiovascular disease). In particular, the meta-analysis showed that long-term compliance is associated with weight loss and a reduction in FBG. Accordingly, LGID interventions are recommended to last for at least four weeks, especially if body mass and FBG are used as outcomes.

## Figures and Tables

**Table 1:** Basic characteristics of the included studies.

First Author (Year)	Country	Diabetes Type	*N*	*N* Int.	*N* Con.	Gender Ratio (F:M)	BMI	Average Age (Year)	FBG (mmol/L)
Jenkins, D.J (2008) [27]	Canada	T2DM	210	106	104	0.6	30.9	60.5	NS
Turner-McGrievy (2011) [28]	USA	T2DM	99	49	50	1.5	34.8	55.2	NS
Ebbeling, C.B (2003) [29]	USA	adiposity	14	7	7	2.2	36.0	16.1	NS
Wolever, T.M (2008) [30]	Canada	T2DM	162	56	106	1.2	31.0	59.9	7.4
Wolever, T.M (2008) [31]	Canada	T2DM	135	46	89	1.2	31.0	59.7	7.3
Malin, S.K (2012) [32]	USA	MetS	21	11	10	0.9	35.5	66.2	8.0
Kelly, K.R (2011) [33]	USA	adiposity	28	13	15	1.2	34.2	66.0	5.9
Mulya, A (2017) [15]	USA	adiposity	19	9	10	0.9	34.0	64.0	5.5
Osella, A.R (2018) [34]	USA	MetS	231	55	176	0.7	NS	57.6	NS
Gomes, J.M (2017) [35]	Brazil	T2DM	20	10	10	1.0	29.2	42.4	8.2
Solomon, T.P (2009) [36]	USA	adiposity	32	15	17	1.1	33.8	66.0	5.6
Fraser, A (2008) [37]	Israel	T2DM	201	73	128	1.5	31.4	56.0	10.3
Visuthranukul, C (2015) [38]	Thailand	adiposity	52	25	27	0.5	33.6	12.0	5.0
Jenkins, D.J (2011) [39]	Canada	T2DM	152	79	73	0.6	30.5	61.5	NS
Pavithran, N (2020) [40]	India	T2DM	36	18	18	0.7	27.0	52.0	NS
Solomon, T.P (2013) [41]	USA	adiposity	20	10	10	1.2	35.0	65.0	5.9
Fabricatore, A.N (2011) [42]	USA	T2DM	79	40	39	3.9	36.3	52.7	NS
Argiana, V (2014) [43]	Greece	T2DM	58	28	30	1.1	32.5	62.2	NS
Li, D (2014) [44]	China	T2DM	54	36	18	0.6	24.3	56.0	6.8
Mirza, N.M (2011) [45]	USA	adiposity	88	45	43	0.9	29.5	12.2	NS
Hu, Z.G (2014) [16]	China	GDM	140	66	74	All famale	NS	30.0	5.0
Heilbronn, L.K (2002) [17]	Australia	T2DM	45	24	21	1.0	33.8	56.7	7.0
Basharat, S (2018) [46]	Pakistan	adiposity	74	38	36	All famale	31.4	27.0	NS
Abete, I (2008) [14]	Spain	adiposity	32	16	16	0.8	32.5	36.0	NS

Int. = Intervention group; Con. = Control group; T2DM = type 2 diabetes mellitus; MetS = metabolic syndrome; GDM = gestational diabetes mellitus; NS = not stated; FBG = fasting blood glucose. RCT-related characteristics of the included studies.

**Table 2:** RCT-related characteristics of the included studies.

First Author (Year)	Time Duration	Study Length	Randomization Method	Blinding	Energy Restriction	Int. Type	Con.Type	GI Int.	GI Con.	CHO(%) Int.	CHO(%) Con.
Jenkins, D.J (2008)	8 July 2004–22 May 2007	6 months	ID	Yes	No	LGID	HCFD	69.6	83.5	44.0	47.5
Turner-McGrievy (2011)	2003–2004	22 weeks	NS	No	No	LGID	ADAD	NS	NS	NS	NS
Ebbeling, C.B (2003)	1 December 2000–30 September 2001	6 months	NS	No	No	LGID	LFD	58.0	59.0	58.0	52.0
Wolever, T.M (2008)	NS	1 year	Opaque envelope	No	No	LGID	HGID/LCHOD	55.1	61.3	51.9	43.1
Wolever, T.M (2008)	January 2002–October 2003	1 year	Opaque envelope	No	No	LGID	HGID/LCHOD	55.0	61.4	52.0	42.7
Malin, S.K (2012)	NS	12 weeks	NS	No	No	LGID	HGID	40.3	80.3	56.2	57.8
Kelly, K.R (2011)	NS	12 weeks	NS	No	No	LGID	HGID	40.3	80.2	55.8	57.7
Mulya, A (2017)	NS	12 weeks	NS	No	No	LGID	HGID	40.0	80.0	NS	NS
Osella, A.R (2018)	December 2007–April 2008	24 weeks	Nonce	Yes	No	LGID	Med-D/LGIMD/RD	NS	NS	46.5	NS
Gomes, J.M (2017)	April 2007–September 2007	30 days	NS	Yes	No	LGID	HGID	35.8	74.1	59.8	53.5
Solomon, T.P (2009)	NS	7 days	NS	No	No	LGID	HGID	41.1	80.9	56.2	57.2
Fraser, A (2008)	March 2003–April 2004	12 months	Nonce	Yes	Yes	LGID	MMD/ADA	NS	NS	NS	NS
Visuthranukul, C (2015)	January 2010–January 2013	6 months	NS	No	No	LGID	LFD	NS	NS	NS	NS
Jenkins, D.J (2011)	May 2004–May 2007	6 months	ID	Yes	No	LGID	HCFD	69.6	83.5	44.0	47.5
Pavithran, N (2020)	October 2018–April 2019	24 weeks	NS	No	No	LGID	RD	NS	NS	NS	NS
Solomon, T.P (2013)	NS	3 months	NS	No	No	LGID	HGID	39.8	80.0	54.7	55.6
Fabricatore, A.N (2011)	September 2006–July 2009	40 weeks	Nonce	No	Yes	LGID	LFD	63.6	63.4	44.0	46.0
Argiana, V (2014)	NS	12 weeks	Nonce	No	Yes	LGID	RD	19.0–49.0	60.0–65.0	NS	NS
Li, D (2014)	June 2013–October 2013	12 weeks	NS	No	No	LGID	RD	NS	NS	54.6	52.1
Mirza, N.M (2011)	NS	12 weeks	NS	No	No	LGLD	HGLD	36.1	59.5	NS	NS
Hu, Z.G (2014)	October 2011–April 2013	5 days	NS	No	Yes	LGID	RD	NS	NS	NS	NS
Heilbronn, L.K (2002)	NS	12 weeks	NS	No	Yes	LGID	HGID	43.0	75.0	58.9	60.8
Basharat, S (2018)	April 2005–2005 June	12 weeks	NS	No	No	LGID	RD	44.6	66.0	45.0	52.7
Abete, I (2008)	NS	8 weeks	NS	No	Yes	LGID	HGID	40.0–45.0	60.0–65.0	NS	NS

ADAD = American Diabetes Association Diet; Med-D = Mediterranean diet; LGID = low-glycemic index diet; MMD = modified Mediterranean diet; HGID = high-glycemic index diet; LFD = low-fat diet; LGIMD = low-glycemic index Mediterranean diet; LCHOD = low-carbohydrate diet; HCFD = high-cereal fiber diet; RD = routine diet; LGLD = low-glycemic load diet; HGLD = high-glycemic load diet.

## Data Availability

Not applicable, due to being systematic review with meta-analyses. All data is available in primary studies.
